# Improving effect of oleic acid-mediated sodium caseinate-based encapsulation in an ultrasound field on the thermal stability and bioaccessibility of quercetin

**DOI:** 10.1016/j.ultsonch.2022.106169

**Published:** 2022-09-17

**Authors:** Shengnan Wang, Yunjun Liu, Yixiang Liu, Zixin Guo, Jie Li

**Affiliations:** a College of Ocean Food and Biological Engineering, Jimei University, Xiamen, Fujian 361021, China; b Collaborative Innovation Center of Provincial and Ministerial Co-construction for Marine Food Deep Processing, Dalian Polytechnic University, Dalian 116034, China

**Keywords:** Quercetin, Oleic acid, Sodium caseinate, Ultrasound, Self-assembly, Bioaccessibility

## Abstract

The simultaneous improvement of quercetin (QUE) processing stability and bioavailability has always presented a technical challenge during food processing. This study constructed a water-soluble carrier consisting of oleic acid (OA) and sodium caseinate (NaCas) in an ultrasonic field and investigated the effect of its encapsulation on improving the thermal stability and bioaccessibility of QUE. The results showed that the OA and NaCas generated uniform, stable water-soluble particles with a poly dispersity index (PDI) below 0.3 and an absolute value of Zeta potential above 30 mV in optimized conditions (a protein concentration of 4 mg/mL, ultrasonic power of 300 W, and ultrasonic time of 5 min). OA-NaCas mass ratio of 1:40, 1:15, 1:8, and 1:4 was selected for QUE loading to compare its encapsulation effect at different mass ratios. Compared with the NaCas without OA, the QUE embedding rate reached 95 % at OA-NaCas mass ratios of 1:15 and 1:8. In addition, the transmission electron microscopy (TEM) images confirmed that QUE was embedded in OA-NaCas particles, forming regular, spherical OA-NaCas-QUE particles at mass ratios or 1:15 and 1:8. Next, when heated at 80 °C for 120 min, the OA-NaCas (OA:NaCas, 1:15, 1:8, and W/W) particles significantly improved the QUE retention rate. The simulated in vitro gastrointestinal digestion experiments showed that the QUE bioaccessibility increased from 25 % to more than 60 % when it was encapsulated in OA-NaCas (OA:NaCas, 1:15, 1:8, and W/W) particles. These results indicated that the OA-NaCas complex was suitable as a hydrophilic delivery carrier of fat-soluble polyphenols.

## Highlights

• Stable OA-NaCas complexes were obtained after ultrasonic treatment at 300 W for 5 min.
• The embedding rate of OA-NaCas complex to QUE was over 90%.
• The OA-NaCas-QUE particles showed regular, spherical at mass ratios or 1:15 and 1:8.
• The heat stability of QUE was enhanced by the OA-NaCas complex.
• The bioaccessibility of QUE was over 60% after encapsulation with OA-NaCas particles.

## 1 Introduction

Quercetin (QUE) is a natural dietary flavone widely found in a variety of fruits and vegetables, including onions, blueberries, and tomatoes [1]. QUE displays pharmacological activity, such as antiviral, antioxidant, anti-inflammatory, anti-obesity, and anti-cancer properties [2], [3], [4]. Despite its beneficial effect, QUE shows poor water solubility of only 0.01 mg/mL (25 °C) [5], and oral absorption and utilization rates in aqueous solutions below 4 % [6], limiting its application in food. In addition, QUE displays poor chemical stability and is highly sensitive to temperature, pH, and metal ions, reducing its biological activity [7]. Therefore, to improve the bioavailability and stability of QUE, different types of packaging systems are examined, including liposomes [8], nanoparticles [9], micelles [10], and inclusion complexes [11].

In recent years, protein carriers have attracted increasing attention due to their biodegradability, environmental friendliness, and renewability [12]. Casein is the main protein in milk, accounting for about 80 %. Due to its high hydrophobicity, a single casein can be stabilized by forming a micelle structure [13], which consists of four caseins (α_s1_, α_s2_, β, and κ-casein) and amorphous calcium phosphate with α_s1_, α_s2_, and β-casein located inside the micelles and κ-casein on the surfaces, spatially stabilizing the micelles [14], [15]. Due to hydrophobic interaction, the related casein clusters or fragments are evenly distributed throughout the micelle structure, playing a crucial as the binding site for hydrophobic bioactive substances [13]. Casein is used in the assembly of fat-soluble polyphenols. For example, in aqueous solutions, sodium caseinate (NaCas) displays a strong affinity to resveratrol and can be used as a carrier [16]. The embedding rate of QUE and curcumin encapsulated by NaCas reached 90 %, while their chemical stability was also improved [17]. NaCas displays a good binding ability to small molecules, as well as excellent stability and self-assembly properties [14], [18]. Based on the previous research in our laboratory, oleic acid (OA) can form water-soluble particles with bovine serum protein, which can improve the stability and bioavailability of fat-soluble active substances [19]. In addition, OA binds tightly to casein and increases its hydrophobicity [20]. Therefore, preparing a NaCas-OA complex is feasible for encapsulation and delivery of bioactive compounds.

Ultrasound has been proven an effective tool for encapsulating active molecules and improving the efficiency and stability of protein encapsulation. For example, Liang et al. used ultrasonic treatment to obtain compact, uniform, and spherical QUE-casein phosphopeptide-chitosan composite nanoparticles, while improving the stability and antioxidant activity of QUE in the processing and gastrointestinal digestive environment [21]. Hui Jiang et al. ultrasonically treated whey protein and garlicin at 50 W/L for 20 min, improving their binding ability, as well as the stability and emulsification properties of garlicin [22]. These results suggest that ultrasonic treatment may effectively improve the interaction between protein and fat-soluble polyphenols. Previous studies have shown that lipids can promote the absorption of fat-soluble polyphenols compared with protein-based packaging [23]. Compared with bovine serum albumin without OA, the addition of OA improves the stability and bioavailability of fat-soluble carotenoids [23]. Therefore, it is reasonable to speculate ultrasound-assisted OA-mediated self-assembled QUE-NaCas promotes the stability and bioaccessibility of QUE.

This study mainly aims to establish hydrophilic OA-NaCas complex particles and improve QUE stability and bioavailability in an aqueous environment. First, the optimal conditions for the formation of OA-NaCas are established at different protein concentrations, ultrasonic power levels, and ultrasonic times. Second, the effect of OA-NaCas complexes on the physicochemical properties of QUE at different mass ratios is compared. Finally, the stability and bioaccessibility of OA-NaCas complexes to QUE at different mass ratios are explored. This study provides an effective strategy for simultaneously improving the stability and bioaccessibility of fat-soluble polyphenols in water and is highly significant for solving the application challenges of fat-soluble polyphenols in hydrophilic food systems.

## 2 Materials and methods

### 2.1 2.1Materials

The NaCas (99 % purity, CAS# 9005-46-3) was purchased from West Asia Chemical Industry Co., Ltd. (Shangdong, China), while the OA (≥98 % purity) was obtained from Sinopharm Chemical Reagent Co., Ltd. (Beijing, China). The QUE (≥98 % purity, CAS#117-39-6) was purchased from Solarbio Technology Co., Ltd. (Beijing, China), while the pepsin (3000.0 U/mg protein) and pancreatin (from porcine pancreas, 259.0 U/mg protein) were obtained from Aladdin Biochemical Technology Co., Ltd. (Shanghai, China). The hydrochloric acid, sodium hydroxide, ethanol, and other chemicals were of analytical grade and obtained from Sinopharm Chemical Reagent Co., Ltd. (Beijing, China).

### 2.2 Preparation of the OA-NaCas particles

The OA-NaCas particles were prepared using a previously described method with slight modifications [19]. Briefly, 2.0 g, 1.6 g, 1.2 g, 0.8 g, 0.4 g, and 0.2 g of NaCas powder was dissolved in 200 mL ultra-pure water, stirred with a magnetic stirrer for 30 min, and placed in a 4 °C refrigerator overnight to reach full hydration, after which respective NaCas solutions of 10 mg/mL, 8 mg/mL, 6 mg/mL, 4 mg/mL, 2 mg/mL, and 1 mg/mL were prepared. Then, an OA ethanol solution was added to obtain OA-NaCas mass ratios of 1:40, 1:20, 1:15, 1:10, 1:8, 1:5, 1:4, 1:3, and 1:2 respectively, with an ethanol level of 1 % (v/v). After stirring for 5 min using a magnetic stirrer, the mixture was kept in the dark for 30 min to react. Then, 4 mg/mL of the NaCas solution was used to further explore the effect of ultrasonic conditions on the formation of OA-NaCas particles. Next, 10 mL of the NaCas solution was placed in a glass bottle, after which different OA quantities were added according to the above mass ratios. The OA-NaCas samples were ultrasonically treated for 5 min using an ultrasonic cell disruptor (Ningbo Xinzhi Biotechnology Co., Ltd., China) at 100 W, 300 W, and 500 W, respectively. The ultrasonic power was fixed at 300 W, and the OA-NaCas solution was ultrasonically treated for 3 min, 5 min, and 7 min, respectively. All the samples were placed in an ice water bath to avoid excessive temperatures and left to react in the dark for 30 min.

### 2.3 Preparation of the OA-NaCas-QUE particles

Different quantities of OA were added to the NaCas solution to obtain OA-NaCas mass ratios of 1:40, 1:15, 1:8, and 1:4. Then, a QUE ethanol solution (5 mg/mL) was added, and the OA-NaCas-QUE solution was ultrasonicated for 5 min at 300 W. The mixture was then placed in the dark for 30 min to fully react. The final QUE concentration in the solution was 0.025 mg/mL, while a NaCas-QUE solution without OA was used as the control.

### 2.4 Particle size and Zeta-potential

The dynamic light scattering technique was used to characterize the particle size and Zeta potential of self-assembled particles. As described in a previous method [24], Zetasizer Nano-ZS90 (Malvern Instruments, Worcestershire, UK) was used to measure the particle sizes and Zeta potential of samples at a fixed scattering angle of 90° and a temperature of 25 °C. Each sample was evaluated three times.

### 2.5 Encapsulation efficiency (EE) of the QUE

The packaging efficiency was measured according to a previously delineated method with some modifications [25]. The samples were subjected to an ultrafiltration centrifugal filtration device (MW intercepting 10 kDa, Beijing Lanjieke Technology Co., Ltd., China) to separate the free (unloaded) QUE from that loaded in the nanocomposites. After the sample was centrifuged at 9000 rpm for 30 min, the free QUE was filtered into a centrifugal tube, while the complex loaded with QUE was intercepted in the device. The absorbance of the centrifuge solution was measured at 370 nm, while the free QUE concentration was determined via the standard curve drawn according to the standard QUE solution. The packaging efficiency of the QUE was calculated using the following equation:

(1) $$\mathrm{EE}\%=\frac{Total\;quercetin-Free\;quercetin}{Total\;quercetin}\times 100\%$$

### 2.6 Transmission electron microscopy (TEM)

The prepared sample solution was diluted to 0.2 mg/mL, dropped onto a 230-mesh copper grid coated with carbon, and air-dried in a dark environment. The samples were observed via TEM (H-7605, Hitachi Co., Ltd., Japan) at 100.0 kV.

### 2.7 The thermal stability of OA-NaCas-QUE

The OA-NaCas-QUE particles were heated in a water bath at 80 °C for 20 min, 40 min, 60 min, 80 min, and 120 min, respectively, to measure their thermal stability. The heated samples were mixed with an ethanol solution at a ratio of 1:4 (V:V), while the sample was eddy shaken for 5 min and then centrifuged at 5000 rpm for 10 min. The supernatant was collected, and the QUE content was determined via High Performance Liquid Chromatography (HPLC).The retention rate of QUE was calculated using the following equation:

(2) $$Rentention\;rate\;\%=\frac{M_{t}}{M_{0}}\times 100\%$$

where *M_t_* denotes the remaining quantity of QUE in the solution, *M_0_* refers to the QUE content in the solution.

### 2.8 Simulated gastrointestinal digestion

Simulated stomach digestion: The simulated gastric fluid (SGF) (2.0 g/mL NaCl and 3.2 mg/mL trypsin with a pH of 2.0) was mixed with the sample at a 1:1 (V:V) ratio. The pH of the mixture was adjusted to 2.0 using 0.1 mol/L HCl, followed by culturing on a shaker (100 rpm) at 37 °C for 2 h. Then, the digested gastric juice was cooled in ice water for 10 min, and the pH was adjusted to 7.0 with 1.0 mol/L NaOH.

Simulated intestinal digestion: The digested gastric fluid and simulated intestinal fluid (SIF) (1.0 mg/mL trypsin and 20.0 mg/mL bile salt with a pH of 7.4) were mixed at a ratio of 1:1 (V:V). The pH of the mixture was adjusted to 7.4 using 0.1 mol/L NaOH and cultured on a shaking table (100 rpm) at 37 °C for 2 h, after which the digested intestinal juice was cooled in ice water for 10 min.

The digested gastric and intestinal fluid was collected and mixed with an ethanol solution at 1:4 (V:V), vortexed for 5 min, and centrifuged at 5000 rpm for 10 min. The supernatant was collected, and the QUE content was measured via HPLC. The retention rate of QUE in gastric and intestinal juice was calculated using the following equation:

(3) $$Rentention\;rate\%=\frac{M_{t}}{M_{0}}\times 100\%$$

where *M_t_* denotes the remaining quantity of QUE in the solution, *M_0_* represents the initial QUE content in the solution.

The digested gastric fluid and intestinal fluid were collected and centrifuged at 5000 rpm for 15 min. The supernatant was collected and diluted with ethanol, after which the QUE content was determined via HPLC. The QUE content in intestinal fluid was calculated using the following equation:

(4) $$Bioaccessibility\;\%=\frac{M_{\mathrm{micelle}}}{M_{0}}\times 100\%$$

where *M_micelle_* represents the remaining quantity of QUE in the micelles, *M_0_* denotes the initial QUE content in the solution.

### 2.9 Determination of QUE via HPLC

According to the previous studies [26], [27], the QUE was analyzed by using HPLC. The prepared samples were filtered using a 0.22 um organic microporous filter membrane and stored at 4 °C in a refrigerator until further use. An Agilent 1260 series HPLC with a diode array detector and a Merck Purospher STAR RP-C18 column (250.0 mm × 4.6 mm, 5.0 μm) (Merck, Darmstadt, Germany) were used for the HPLC analysis using a mobile phase consisting of 70 % methanol and 30 % water (containing 0.1 % acetic acid) at a flow rate of 0.8 mL/min, a column temperature of 30 °C, and an injection volume of 20 uL. The chromatographic QUE signal was recorded at 370 nm, while the QUE concentration was determined according to the peak area and the standard curve.

### 2.10 Statistical analysis

All the measured values were carried out three times and SPSS (version 24.0, SPSS Inc., Chicago, IL, USA) was used for one-way of variance (ANOVA) and Duncan's multiple comparisons. The data were expressed as mean ± standard deviation. The significance was set as *P* < 0.05.

## 3 Results and discussion

### 3.1 The effect of the OA-NaCas mass ratio on the physical and chemical properties of the OA-NaCas particles

**Fig. 1:**
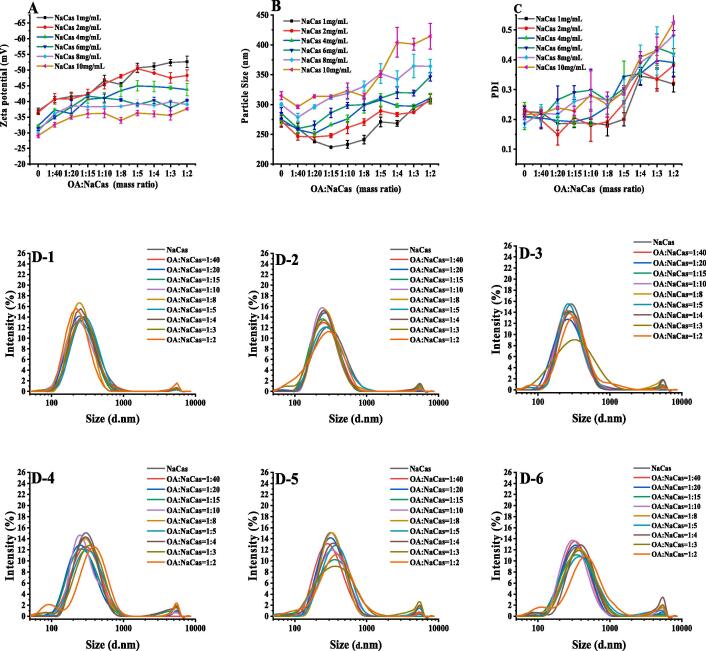
(A) Zeta potential. (B) Particle size. (C) PDI. (D) The OA-NaCas size distribution (D-1 to D-6: 1 mg/mL, 2 mg/mL, 4 mg/mL, 6 mg/mL, 8 mg/mL, and 10 mg/mL).

This study examined the effect of different NaCas protein concentrations and OA to NaCas mass ratios on the potential, particle size, and particle size distribution of the OA-NaCas complexes. As shown in Fig. 1A-B, compared with the NaCas without OA, the addition of OA can significantly improve the Zeta potential of the system while changing the particle size at the same time. Within the selected OA concentration range (0.025–2.00 mg/mL), the Zeta potential of OA-NaCas complexes gradually decreased in conjunction with higher protein concentrations, while the particle sizes slowly declined. At an OA-NaCas (W:W) ratio of 1:4, the NaCas concentration gradually increased from 1 mg/mL to 10 mg/mL, the Zeta potential of the OA-NaCas complex decreased from −51.16 ± 1.01 mV to −35.96 ± 0.95 mV, and the particle sizes increased from 268.23 ± 4.76 nm to 404.16 ± 24.82 nm. In a protein concentration range of 1–10 mg/mL, a single peak was evident for the particle size distribution of the OA-NaCas complex, indicating that the OA and NaCas formed a uniform dispersion system via self-assembly (Fig. 1D1-D6). Research has shown that the system is more stable with smaller particle sizes when the absolute Zeta potential value of the hydrophilic particles is large [28]. Therefore, considering the potential, particle sizes, and particle size distribution parameters of the preparation system, a NaCas concentration of 4 mg/mL was selected for the subsequent experiments.

The OA concentration significantly affects the physicochemical properties of the OA-NaCas complex. At a NaCas concentration of 4 mg/mL, the OA-NaCas (W:W) ration increased from 1:40 to 1:10, while the Zeta potential rose from −32.26 ± 0.28 mV to −41.10 ± 1.63 mV, and the particle size slowly increased from 251.90 ± 4.29 nm to 276.10 ± 6.46 nm. When the OA proportion was further increased, the OA-NaCas (W:W) ratio ranged between 1:8–1:2, while the Zeta potential and particle size of the OA-NaCas complex remained basically unchanged at about −45 mV and 300 nm, respectively. This could be attributed to a large number of cavities and channels in the internal structure of the natural NaCas micelles [29]. When the OA content is low, it may be embedded into the cavities of the NaCas micelles. The interaction between the two changed the NaCas structure, reducing the sizes of the OA-NaCas particles. An increase in the OA content allowed it to fill the cavities inside NaCas micelles. Furthermore, high OA content inhibited the ability of NaCas to bind with OA, causing the remaining OA to adhere to the NaCas surface, increasing the sizes of the OA-NaCas particles.

### 3.2 The effect of ultrasonic treatment on the formation of OA-NaCas particles

#### 3.2.1 The effect of ultrasonic power on the formation of OA-NaCas particles

**Fig. 2:**
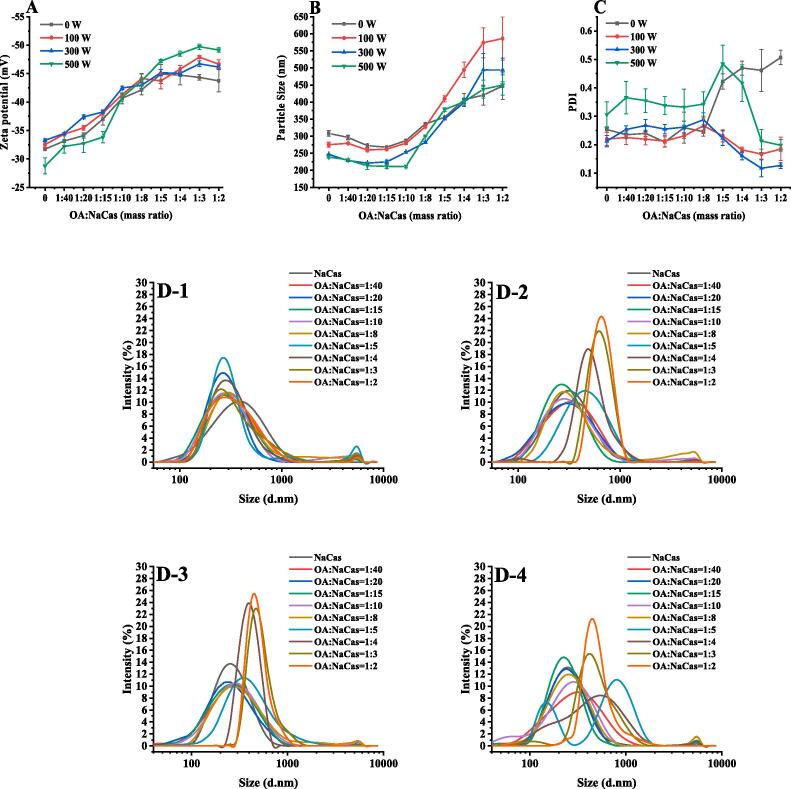
(A) Zeta potential. (B) Particle size. (C) PDI. (D) OA-NaCas size distribution (D-1 to D-4: 0 W, 100 W, 300 W, and 500 W).

Ultrasonic treatment increases the interaction with small molecules by changing the protein structures [30]. Therefore, this study examined the effect of ultrasonic treatment on the OA-NaCas self-assembly at a NaCas concentration of 4.0 mg/mL (Fig. 2A-B). The Zeta potential of the OA-NaCas complex did not change significantly when subjected to 100 W for 5 min at an OA-NaCas (W:W) ratio ≤ 1:4 but increased slightly at an OA-NaCas (W:W) ratio of 1:3 or 1:2. At an OA-NaCas (W:W) ratio ≥ 1:5, the particle size of the complex increased significantly. Increasing the ultrasonic power at the same ultrasonic time changed the situation dramatically. When the ultrasonic power was increased to 300 W, the Zeta potential of the OA-NaCas complex increased to varying degrees, while the particle size (OA-NaCas ≤ 1:8, W:W) decreased significantly from 296.25–335.86 nm to 228.74–281.92 nm. When the ultrasonic power was further increased to 500 W, the Zeta potential of the complex decreased slightly at a low OA concentration (OA-NaCas ≤ 1:10, W:W), but when OA-NaCas (W:W) ≥ 1:5, the Zeta potential of the complex increased and even reached −49.70 mV (OA-NaCas = 1:3, W:W). However, the particle size of the composite did not decrease significantly with increased ultrasonic power. As shown in the particle size distribution diagram (Fig. 2D1-D4) showed a more uniform particle size distribution in the NaCas with a higher OA load due to ultrasonic treatment compared with that of the OA-NaCas complex without ultrasound exposure. Based on the potential, particle size, and particle size distribution parameters of the composite, an ultrasonic power of 300 W was selected for subsequent experiments.

#### 3.2.2 The effect of ultrasonic time on the formation of OA-NaCas particles

**Fig. 3:**
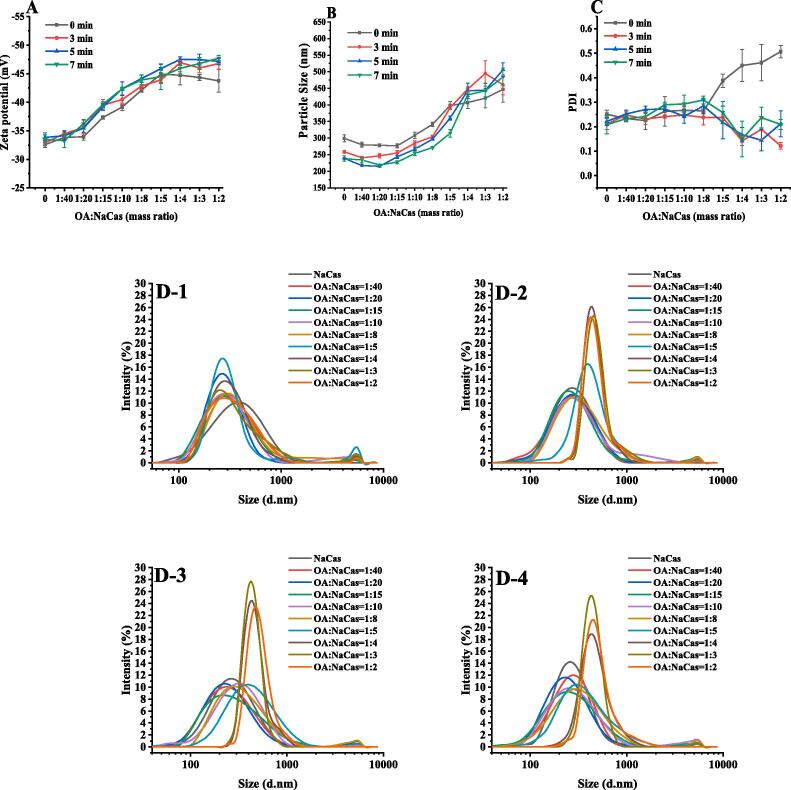
(A) Zeta potential. (B) Particle size. (C) PDI. (D) OA-NaCas size distribution (D-1 to D-4: 0 min, 3 min, 5 min, and 7 min).

Ultrasonic treatment facilitates mechanical mass transfer, heating, and cavitation, providing more opportunities for NaCas to bind small hydrophobic molecules [31]. Therefore, this study examined the influence of different ultrasonic times on the interaction between OA and NaCas at 300 W. As shown in Fig. 3, compared with the OA-NaCas complex without ultrasonic treatment, the absolute Zeta potential value of the complex after ultrasonic treatment increased, while the particle size decreased. This result was consistent with earlier studies: the absolute value of the Zeta potential increased by 11 mV while the particle size decreased from 108.65 nm to 18.62 nm after ultrasonic treatment of rice bran protein and chlorogenic acid complexes [30]. The results indicated that the ultrasonic treatment time affected the OA-NaCas interaction. When OA-NaCas (W:W) = 1:8, the particle size of the OA-NaCas complex obtained without ultrasonic treatment was 341.40 ± 6.02 nm, decreasing to 303.91 ± 4.95 nm and 271.55 ± 2.28 nm at 3 min and 7 min respectively, with a slight Zeta potential increase (from −42.12 mV to −43.92 mV). In addition, ultrasonic treatment rendered the particle size distribution of the composite more uniform, especially in the case of a relatively large OA ratio (OA-NaCas, 1:5–1:2, W:W). At an OA-NaCas (W:W) ratio = 1:4, the PDI value of the system decreased from 0.45 ± 0.06 to 0.14 ± 0.01 after 3 min of ultrasound exposure. Considering the changes in the physical and chemical properties of the OA-NaCas complex, an ultrasonic time of 5 min was selected for the subsequent experiments. Furthermore, to compare the QUE packaging differences in the OA-NaCas complexes with varying OA levels, OA-NACas mass ratios of 1:40, 1:15, 1:8, and 1:4 were selected for the subsequent packaging experiments.

### 3.3 Examining the QUE embedding effect of the OA-NaCas complex

#### 3.3.1 The physicochemical properties of the OA-NaCas-QUE complex

**Fig. 4:**
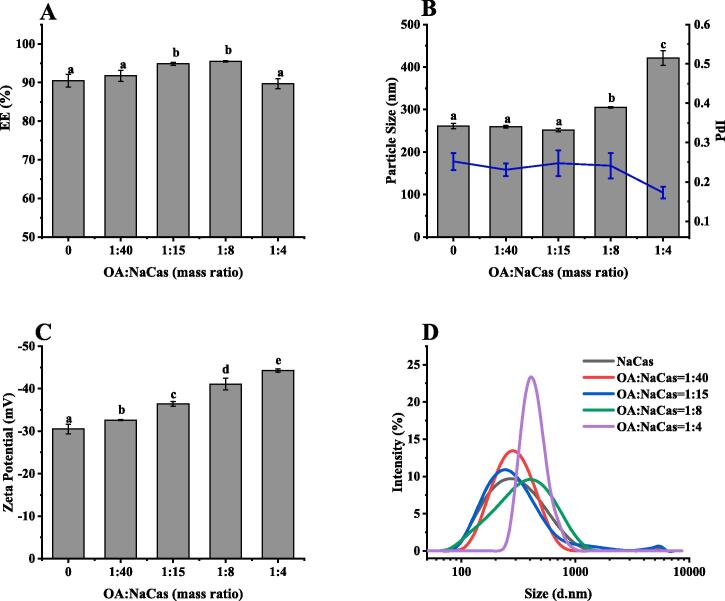
(A) The EE of QUE, (B) particle size and PDI, (C) Zeta potential, and (D) size distribution of the OA-NaCas-QUE.

As shown in Fig. 4A, an OA-NaCas mass ratio of 1:15 and 1:8 further improved the QUE encapsulation rate from 90 % to about 95 % compared with NaCas. However, the encapsulation rate of the OA-NaCas complex to QUE decreased at a higher OA ratio (OA-NaCas, 1:4, W/W). This indicated that the embedding efficiency of QUE varied in conjunction with the OA level. Moreover, different OA concentrations influence the particle size, Zeta potential, and particle size distribution of the OA-NaCas-QUE complex differently (Fig. 4B-D). At OA-NaCas mass ratios of 1:8 and 1:4, the particle size of OA-NaCas-QUE increased from 260.63 nm to 304.74 nm and 421.07 nm, respectively, compared with NaCas-QUE. In addition, the Zeta potential of the OA-NaCas-QUE complex increased at a higher OA ratio. When the OA mass ratio increased from 1:40 to 1:4, the Zeta potential of the QUE complex increased from −32.64 mV to −44.22 mV, indicating that OA played an essential role in regulating the surface charge of the OA-NaCas-QUE complex [23]. From the perspective of particle size distribution, the NaCas-QUE and OA-NaCas-QUE particles with different mass ratios formed a single peak, while the PDI values are all < 0.3, indicating that the prepared particles formed a uniform dispersion system in aqueous solutions.

#### 3.3.2 TEM observation of the OA-NaCas-QUE complex

**Fig. 5:**
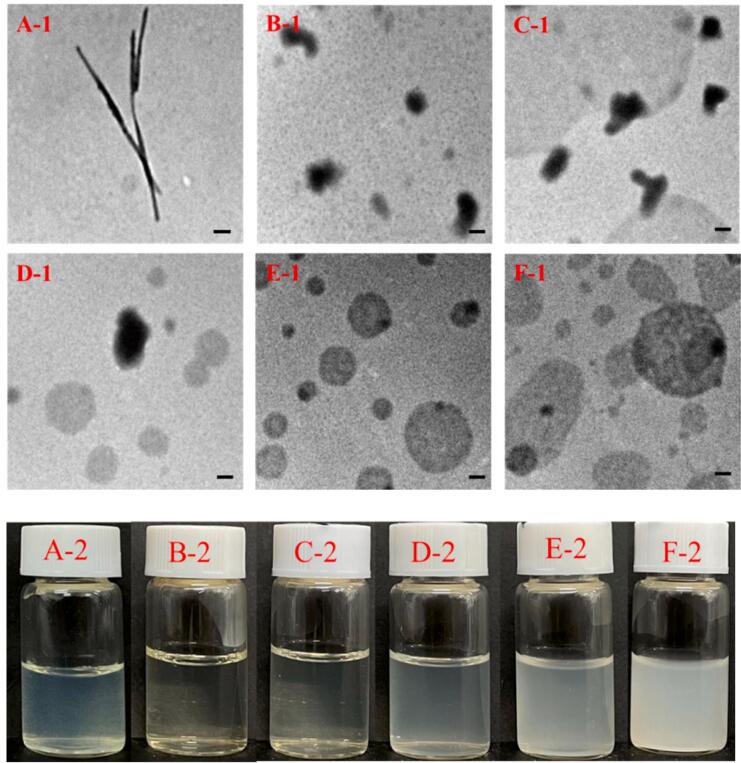
(A-1) and (A-2) represent the transmitted image and visual appearance of quercetin in water, respectively. ((B-1-E-1) and (B-2-E-2) represent the transmitted image and visual appearance of QUE-OA-NaCas particles at OA-NaCas mass ratios of 0, 1:40, 1:15, 1:8, and 1:4, respectively (Scale bar = 0.1 μm).

Since QUE displayed exceedingly low water solubility, the QUE ethanol solution formed a precipitate when added to water. The morphology of these sediments (Fig. 5A-1) exhibited typical needlelike QUE crystals [30]. Fig. 5B-1 shows the TEM image of NaCas-QUE. The needlelike QUE completely disappeared after the QUE was wrapped by NaCas, indicating that NaCas effectively encapsulated the QUE, causing the particles to present irregular shapes. At an OA-NaCas mass ratio of 1:40, the shape of OA-NaCas-QUE remained basically unchanged compared with that of NaCas-QUE (Fig. 5C-1). When the mass ratio increased to 1:15 and 1:8, OA-NaCas-QUE exhibited a regular spherical shape (Fig. 5D-1-E-1). When OA content increased further, OA-NaCas-QUE began to aggregate into large particles.

### 3.4 The stability of the OA-NaCas-QUE complex

#### 3.4.1 Thermal stability of QUE

**Fig. 6:**
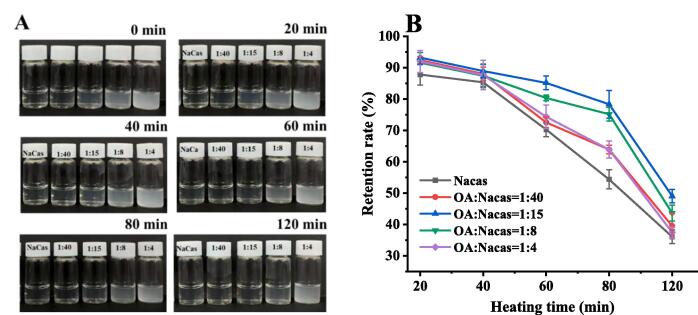
(A) The visual appearance changes (B) the retention rates and of QUE in the NaCas, OA-NaCas mass ratios of 1:40, 1:15, 1:8, and 1:4 particles when heated at 80 °C from 0 min to 120 min.

Heat treatment accelerates the oxidative degradation of QUE during food processing and storage [32]. Since QUE is difficult to dissolve in water and forms precipitate, as mentioned in Section 3.3.2, it cannot be accurately measured. Therefore, this study only evaluated the influence of different encapsulation conditions on the thermal stability of QUE. As shown in Fig. 6, when the complex was heated at 80 °C, the appropriate addition of OA improved the retention rate of QUE compared with the NaCas carrier. At an OA-NaCas (W:W) ratio of 1:15 or 1:8, the QUE retention rate remained above 80 % after heating the compound for 80 min. Contrarily, at an OA/NaCas (W:W) ratio of 1:40 or 1:4, QUE displayed a retention rate of only 65 %. This may be because the OA-NaCas-QUE particles are relatively regular and spherical at an OA-NaCas (W:W) ratio of 1:10 or 1:8 (Fig. 5) with a small heating area. Therefore, QUE is not prone to oxidative degradation. In addition, the OA-NaCas-QUE particles did not settle and flocculate when heated at 80 °C for 20–120 min (Fig. 6A).

### 3.5 The bioaccessibility of QUE

**Fig. 7:**
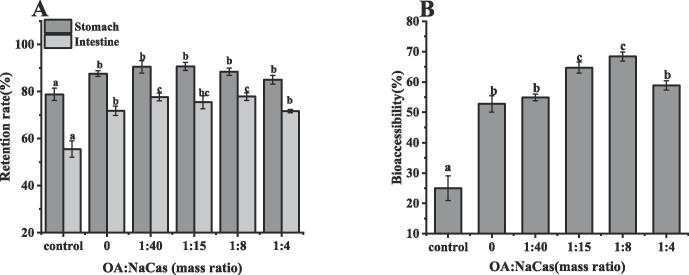
The retention rate and bioaccessibility of QUE after the simulated gastrointestinal digestion. (A) Retention rate. (B) Bioaccessibility (Control represents the free QUE). Different lowercase letters indicate significant differences (*P* < 0.05).

In this study, the stability and bioaccessibility changes of free QUE, NaCas-QUE, and OA-NaCas-QUE during gastrointestinal digestion were observed by simulating the gastrointestinal digestion model in vitro. As shown in Fig. 7A-B, after 2 h of simulated digestion of gastric fluid, about 78 % of free QUE remained, which may be because the simulated digestion was carried out in the environment and the gastric fluid contained some oxygen, resulting in its oxidative degradation [33]. After digestion by the intestinal fluid for 2 h, the retention rate of QUE decreased significantly, yielding only 55.27 ± 3.34 %, while the bioaccessibility of the free QUE was only 25.15 ± 4.04 % after gastrointestinal digestion in vitro. After encapsulation by NaCas particles, the QUE level in the gastrointestinal environment improved, while the gastric retention rate increased to 87.56 ± 1.11 %, and the intestinal retention rate increased to 71.71 ± 1.96 %. Adding an appropriate amount of OA further improved the QUE stability during the gastrointestinal digestion process. At an OA/NaCas (W:W) ratio of 1:8, the gastric retention rate was 88.38 ± 1.57 %, and the intestinal retention rate was 77.84 ± 1.67 %. This indicates that OA-NaCas particle encapsulation alleviates QUE degradation in the gastrointestinal environment. Furthermore, compared with free QUE, NaCas and OA-NaCas (W:W) are 1:40–1:4 complexes that increased the bioaccessibility of QUE after gastrointestinal digestion by 27 %, 29 %, 39 %, 43 %, and 33 %, respectively. At OA-NaCas mass ratios of 1:15 and 1:8, OA-NaCas-QUE was superior at improving QUE bioaccessibility. Previous studies also showed that adding fatty acids increased the dissolution of lipophilic polyphenols in mixed micelles, improving their bioaccessibility [34].

## 4 Conclusions

This study successfully constructs a fatty acid protein carrier via ultrasonic treatment. The self-assembly of NaCas and OA is affected by the protein concentration, OA-NaCas mass ratio, and ultrasonic treatment conditions. Uniform, stable, water-soluble OA-NaCas particles are formed in optimized conditions (a protein concentration of 4 mg/mL, ultrasonic power of 300 W, and ultrasonic time of 5 min). Measurements of the embedding rate of OA-NaCas-QUE when exposed to different OA levels and TEM observation indicate that QUE is embedded in the OA-NaCas particles. At OA-NaCas (W:W) ratios of 15:1 and 8:1, the OA-NaCas-QUE complex forms regular spherical particles. Furthermore, OA-NaCas (W:W) at ratios of 15:1 and 8:1 can better protect QUE from thermal degradation and improve its bioaccessibility. OA-NaCas particles with appropriate OA levels can enhance the stability and bioaccessibility of fat-soluble polyphenols in a hydrophilic environment. However, further in vivo experiments are necessary to better understand the effect of OA-NaCas particles on QUE absorption.

## CRediT authorship contribution statement

**Shengnan Wang:** Conceptualization, Data curation, Formal analysis, Writing – original draft. **Yunjun Liu:** Methodology, Resources, Software. **Yixiang Liu:** Funding acquisition, Supervision, Writing – review & editing, Project administration. **Zixin Guo:** Writing – review & editing. **Jie Li:** Writing – review & editing.

## Declaration of Competing Interest

The authors declare that they have no known competing financial interests or personal relationships that could have appeared to influence the work reported in this paper.

## Data Availability

No data was used for the research described in the article.
